# Distinct immunologic patterns of response and resistance to anti-PD-1/PD-L1-based immunotherapy in patients with soft tissue sarcoma

**DOI:** 10.3389/fimmu.2026.1783216

**Published:** 2026-03-05

**Authors:** Brie-Anne Mannah, Fei Yang, Lijia Yu, Howard Gurney, Jean Y. H. Yang, John J. Park, Su Yin Lim

**Affiliations:** 1 Macquarie Medical School, Faculty of Medicine, Health and Human Sciences, Macquarie University, Sydney, NSW, Australia; 2 Melanoma Institute Australia, The University of Sydney, Sydney, NSW, Australia; 3 School of Mathematics and Statistics, The University of Sydney, Sydney, NSW, Australia; 4 Sydney Precision Data Science Centre, The University of Sydney, Sydney, NSW, Australia; 5 Nepean Cancer and Wellness Centre, Nepean Hospital, Sydney, NSW, Australia

**Keywords:** biomarkers, immune checkpoint inhibitors, monocytes, peripheral blood mononuclear cells, single-cell RNA sequencing

## Abstract

**Introduction:**

Chemotherapy remains the standard of care for metastatic soft tissue sarcoma (STS), but clinical benefit is modest. Immune checkpoint inhibitors (ICIs), such as anti-programmed cell death 1 (PD-1) and anti-cytotoxic T-lymphocyte antigen 4 (CTLA-4), have transformed cancer treatment, yet their efficacy in STS is variable and largely confined to undifferentiated pleomorphic sarcoma (UPS) and dedifferentiated liposarcoma (LPS). Reliable biomarkers to predict ICI response in STS are understudied and currently lacking.

**Methods:**

We examined mutation profiles and analysed longitudinal blood samples from STS patients (n=13) treated with anti-PD-1/PD-L1–based therapy to identify molecular features and circulating immune correlates of ICI efficacy. To gain deeper insight, single-cell RNA sequencing was performed on peripheral blood mononuclear cells (PBMCs) from a patient with prolonged stable disease (>6 months).

**Results:**

Complete blood counts and PBMC profiling demonstrated that elevated circulating lymphoid cells were associated with response, whereas enrichment of innate immune populations, particularly neutrophils and monocytes, correlated with non-response. Single-cell RNA sequencing of PBMCs from a patient with prolonged stable disease revealed dynamic shifts in monocyte and CD8 T cell phenotypes and inflammatory signalling pathways, which paralleled radiological tumour regression and subsequent progression.

**Discussion:**

Our findings highlight peripheral immune profiles as candidate biomarkers for predicting and monitoring ICI efficacy in STS. Incorporating these immune markers could refine patient selection, reduce unnecessary toxicity, and support adaptive treatment strategies for patients with this rare and heterogenous cancer.

## Introduction

Soft tissue sarcoma (STS) is a rare and heterogeneous group of mesenchymal malignancies that can develop on any part of the body. Approximately 50% of patients with STS develop metastatic disease, with a median overall survival of only 12–18 months. Despite limited efficacy, chemotherapy remains the standard of care for patients with locally advanced or metastatic STS, typically producing short-lived responses and poor long-term outcomes. For instance, chemotherapy response rates range from 20–30% (1, 2), with median progression free survival of only 5.6–11 months (2).

Immune checkpoint inhibitors (ICIs) targeting programmed cell death 1 (PD-1), PD-ligand 1 (PD-L1), cytotoxic T-lymphocyte antigen 4 (CTLA-4) and lymphocyte activation gene 3 (LAG-3) have transformed the treatment landscape for several cancers (3–5); however, their initial application in STS yielded disappointing results. Early clinical trials with the CTLA-4 inhibitor ipilimumab showed no immunological activity or clinical benefit in synovial sarcoma and paediatric sarcoma (6, 7). Nonetheless, emerging evidence suggests that certain STS subtypes may be more sensitive to ICIs. The PD-1 inhibitor pembrolizumab has shown encouraging activity in undifferentiated pleomorphic sarcoma (UPS) and dedifferentiated liposarcoma (LPS) (8), prompting a trial expansion that included more patients with these subtypes (9). In the SARC028 (NCT02301039) expansion cohort, the objective response rate (ORR) was 23% in patients with UPS and 10% in patients with dedifferentiated LPS treated with pembrolizumab monotherapy, with median progression-free survival (PFS) of 3 months and 2 months, respectively (9). Furthermore, combination therapy with nivolumab and ipilimumab has demonstrated improved clinical benefit compared to nivolumab monotherapy in patients with UPS, leiomyosarcoma, myxofibrosarcoma, and angiosarcoma, with ORR of 16%, median PFS of 4.1 months and median OS of 14.3 months with the combination (10).

Collectively, these findings indicate that patients with specific STS subtypes, particularly UPS and LPS, may derive clinical benefit from ICI therapy. Indeed, overall response rates to ICIs in STS - with a 18% objective response rate reported in the SARC028 study (8) - appear comparable to those observed across other malignancies, with an estimated 12.5% of cancer patients in the United States responding to ICIs (11)). However, there are currently no reliable biomarkers to identify STS patients likely to respond and monitor response to ICIs (reviewed in (12)). The development of predictive biomarkers would not only improve patient outcomes by guiding therapy selection but also avoid unnecessary toxicity and allow for treatment adjustments for non-responders.

In this study, we examined complete blood counts and peripheral blood mononuclear cells (PBMCs) from longitudinal blood samples of STS patients (n=13) before and after treatment with anti-PD-1/PD-L1-based inhibitor therapy. We demonstrate that elevated levels of circulating lymphoid cells are associated with ICI response, while increased levels of innate immune cells, particularly neutrophils and monocytes, are associated with non-response. To further explore immune dynamics, we performed single-cell RNA sequencing on PBMCs from a single STS patient with prolonged stable disease (> 6 months) on anti-PD-1 inhibitor therapy. In this patient, distinct shifts in monocyte phenotypes and inflammatory immune signalling pathways aligned with radiological evidence of tumour response and disease progression. Together, these findings highlight circulating immune cell profiles as candidate biomarkers for predicting and monitoring ICI efficacy.

## Materials and methods

### Patients and samples

This study includes 13 patients with stage IV STS enrolled into early phase clinical trials at Macquarie University Clinical Trials Unit (MQCTU) between 2019 and 2023. Written consent was obtained from all patients and research complied with ethical regulations of each individual trial (NCT03475251, NCT03918278, NCT05200013, NCT04111445, NCT05483530). Patients were treated with a PD-1/PD-L1 inhibitor, alone or in combination with another immunotherapy (**Table 1**) until disease progression or intolerable toxicity that required alternative treatments. Blood samples were collected from patients before treatment (baseline) and at 2–9 weeks, 10–16 weeks, 21–24 weeks, and 33–37 weeks post treatment, processed into peripheral blood mononuclear cells (PBMCs) and stored frozen at -80 °C. Patient samples were acquired with consent from the Macquarie University Cancer Biobank. Ethics approval for sample collection (HREC2793) and use (HREC6755) was obtained from the Macquarie University Human Research Ethics Committee. All procedures were conducted in accordance with institutional guidelines, national regulations and the Declaration of Helsinki.

**Table 1:** Clinicopathologic characteristics of patients with soft tissue sarcoma.

Characteristics	All cohort (n=13)	Responder (n=7)	Non-responder (n=6)
Age^a^, median year (range)	56 (27-76)	49 (27–72)	69 (40–76)
Sex, n (%)			
Male	7 (54)	4 (57)	3 (50)
Female	6 (46)	3 (43)	3 (50)
Sarcoma subtype, n (%)			
Leiomyosarcoma	5 (38)	4 (57)	1 (17)
Undifferentiated pleiomorphic sarcoma	4 (31)	2 (29)	2 (33)
Angiosarcoma	2 (15)	1 (14)	2 (33)
Dedifferentiated liposarcoma	1 (8)		1 (17)
Leiomyosarcoma	1 (8)		
Desmoplastic small round cell sarcoma			
Prior therapy, n (%)			
Yes	11 (85)	5 (71)	6 (100)
No	2 (15)	2 (29)	
Treatment received, n (%)			
Anti-PD-1 monotherapy	7 (54)	4 (57)	3 (50)
Anti-PD-1/PD-L1 combination therapy	6 (46)	3 (43)	3 (50)
Best response^b^, n (%)			
CR	1 (8)	1 (14)	6 (100)
SD	6 (46)	6 (86)	
PD	6 (46)		

a Age at start of trial shown.

b Best response as per RECIST 1.1 criteria.

CR, complete response; SD, stable disease; PD, progressive disease.

Patient demographics and clinicopathologic features, including age, sex, sarcoma subtype, information about prior therapy, type of treatment, objective response, PFS and OS were collected. PFS and OS were calculated from the date of cycle 1 day 1 and the date of progressive disease and death, respectively. Investigator-determined objective response was assessed radiologically with computed tomography (CT) scans at 6 weekly intervals using Response Evaluation Criteria in Solid Tumours (RECIST) 1.1 criteria (13). For this study, clinical responders were classified as patients who achieved a complete response (CR), partial response (PR), or stable disease (SD). Non-responders were characterised as patients with progressive disease (PD).

### Molecular profiling of patients

Genomic data analysed using FoundationOne CDx from the Cancer Molecular Screening and Therapeutics (MoST) study were collected, including information about tumour mutation burden (TMB), microsatellite status, and genetic alterations in *CDK4* (cyclin-dependent kinase 4), *CDK12* (cyclin-dependent kinase 12), *CDKN2A/B* (cyclin-dependent kinase inhibitor 2A and 2B), *TP53* (tumour protein 53), and *RB1* (retinoblastoma protein).

### Complete blood counts

Haematological indices including haemoglobin, white cell count (WCC), and specific counts of neutrophils, lymphocytes, monocytes, eosinophils, basophils, and platelets were collected from Barratt and Smith Pathology for each patient at baseline, and at 3- and 6-weeks post treatment. The neutrophil-to-lymphocyte ratio (NLR) was calculated as the neutrophil count divided by the lymphocyte count. The lymphocyte-to-monocyte ratio (LMR) was calculated as the lymphocyte count divided by the monocyte count. The platelet-to-lymphocyte ratio (PLR) was calculated as the platelet count divided by the lymphocyte count.

### Flow cytometry profiling

Cryopreserved PBMCs were thawed into RPMI media containing 20 mg/ml DNAse I (Sigma-Aldrich, St Louis, MO, USA) and washed with PBS prior to staining. Staining of all samples was performed in flow cytometry buffer (PBS supplemented with 5% Foetal Bovine Serum (FBS, Sigma), 10 mM EDTA, and 0.05% sodium azide). PBMCs were incubated on ice for 30 min with fluorescently labelled antibodies detecting extracellular markers (**Supplementary Table 1**) and Fc block (BD Biosciences, Franklin Lakes, NJ, USA) to prevent non-specific staining. PBMCs were then fixed and permeabilised using the Transcription buffer Fixation/Permeabilisation kit (Thermo Fisher Scientific, Waltham, MA, USA) and stained with fluorescently labelled antibodies against intracellular proteins (**Supplementary Table 1**) plus Fc block in permeabilisation buffer. Live Dead near-infrared (NIR) fixable dye (Thermo Fisher Scientific) was added to exclude non-viable events. PBMCs were washed and samples were acquired on a 5-laser LSR Fortessa X20 flow cytometer (BD Biosciences) and analysed using FlowJo software v10.5 (TreeStar, Ashland, OR, USA). All available events were collected, and samples containing less than 200 viable cells were excluded from the study.

FlowJo v10.10 software (BD Biosciences) was used for data analysis. General gating strategy included forward scatter and side scatter to exclude debris, time parameter to exclude electronic noise, forward scatter and height to exclude doublets and gating on viable cells (**Supplementary Figure 1**). Specific gating strategies are summarised in **Supplementary Table 2**. All immune cell subsets identified through extracellular and intracellular staining were examined as a proportion of live cells unless otherwise indicated.

### Plasma protein profiling

Plasma samples were analysed for expression of 96 cytokines, chemokines, and growth factors using the Human Cytokine/Chemokine 96-Plex Discovery Assay^®^ Array (Cat no. HD96, Eve Technologies, Alberta, Canada) on the Luminex™ 200 system (Luminex, Austin, TX, USA) by Eve Technologies Corporation. Each sample was run in duplicate and absolute protein concentrations were interpolated from a standard curve for each analyte and reported as pg/mL. Heatmap visualisation and correlation analysis were performed with Morpheus (https://software.broadinstitute.org/morpheus).

### Single-cell RNA sequencing

PBMCs from a single patient (Patient 3) with prolonged response (SD of >6 months) to PD-1 inhibitor therapy were collected at baseline and 5-, 12- and 24-weeks post treatment for single-cell RNA sequencing (scRNAseq). Cryopreserved PBMCs were thawed into RPMI media containing 20 mg/ml DNAse I (Sigma-Aldrich) and cell viability assessed using trypan blue (Thermo Fisher Scientific). PBMCs (~1000 cells/µl) were loaded into separate wells of a Chromium 3’ Next GEM Chip G (Cat no. PN-2000177, 10x Genomics, Pleasanton, CA, USA), with a targeted capture of 7000 cells. The single cells were partitioned with barcoded Next GEM Single Cell 3’ Gel Beads v3.1 (Cat no. PN-2000164, 10x Genomics) using the Chromium Controller to generate single-cell beads in emulsion. scRNAseq libraries were prepared using the Next GEM Single Cell 3’ Reagent Kits (v3.1), including the Single Cell 3’ Gel Bead Kit (Cat no. PN-1000129, 10x Genomics) and Single Cell 3’ GEM Kit (Cat no. PN-1000130, 10x Genomics), and the Dual Index Plate TT Set A (Cat no. PN-3000431, 10x Genomics) following manufacturer’s instructions. Single-cell barcoded cDNA libraries were quantified using Qubit (Invitrogen, Carlsbad, CA, USA) and TapeStation 4150 (Agilent, Santa Clara, CA, USA) and sequenced by the Australian Genome Research Facility (AGRF) on an Illumina NovaSeq X (Illumina, approximately 600 million reads per sample). The Illumina DRAGEN BCL Convert pipeline (v07.021.645.4.0.3) was then used to convert Binary Base Call (BCL) files generated by the sequencer into FASTQ files for downstream analysis.

### scRNA-seq data processing and quality control

FASTQ files were processed using the Cell Ranger Single Cell Software Suite (v7.0.1, 10X Genomics). After de-multiplexing and barcode processing, reads were aligned to the human reference genome (GRCh38) and aligned reads were filtered according to the default parameters of Cell Ranger to remove low quality reads. Gene-level counts were generated for each sample. The four PBMC samples were aggregated using Cell Ranger aggr (v7.1.0, 10X Genomics) to produce a single gene-cell matrix for downstream analysis.

We performed quality control using Loupe Browser (v8.0.0, 10X Genomics) to include cells with total Unique Molecular Identifier (UMI) counts between 1,448 and 16,384 (log2: 10.5 and 14), with 512 and 2,500 detected genes per cell, and mitochondrial gene percentage < 15%, resulting in 35,534 cells for downstream analyses.

Data were normalised using the scater R package (v1.36.0) logNormCounts function (14). The top variable genes (ntop = 500 for broad annotation and ntop = 200 for fine annotation) were selected for principal component analysis (PCA), and tSNE embeddings were generated from the PCA results for visualisation.

### Cell type annotation

We used scClassify (15) for cell type annotation with a pretrained model based on publicly available PBMC reference datasets (16). To achieve fine-level annotations, we implemented a two-step scClassify procedure using an Asian PBMC atlas (17). Cells were annotated using a model trained on the level 3 reference dataset (downsampled to 1,000 cells per cell type), then trained using cells with confident annotations from prior annotation. This iterative approach reduced ambiguous or inconsistent labels, and the annotations from the newly trained model were used for downstream analyses. Dot plot for cell type marker gene expression was generated using the function *scDotPlot.* Cell type proportions were quantified at each time point using the *scFeatures* package (18). Changes in cell type composition over time were assessed by examining the variance in cell type proportions across the four time points.

### Single sample gene set enrichment analysis

To investigate immune activation states, we assessed the activity of four key inflammatory Hallmark pathways (INTERFERON_ALPHA_RESPONSE, INTERFERON_GAMMA_RESPONSE, IL6_JAK_STAT3_SIGNALING, and TNFA_SIGNALING_VIA_NFKB) within each cell type using ssGSEA (19). For each time point, ssGSEA scores were derived from pseudobulk gene expression values and visualised using the function *pheatmap*, with values scaled by row (time points) to illustrate temporal trends in pathway activity.

### Statistical analysis

Statistical analyses were performed using GraphPad Prism (v10.2.0). Data are shown as mean ± SD unless otherwise indicated. Statistical analyses are specified in the figure legends. Mann-Whitney test was used to compare between two groups. A mixed-effects analysis with Geisser-Greenhouse correction and Sidak multiple comparison was used to determine differences between three groups. A value of *p* < 0.05 was considered statistically significant. All variables with p<0.05 in univariate analyses was included in the multivariate model. Multivariable logistic regression was performed using the brglm2 R package (v1.0.1), adjusting for covariates included STS subtype and treatment regimen.

## Results

### Clinicopathologic features of patients with soft tissue sarcoma

This study included 13 patients diagnosed with STS enrolled in anti-PD-1 or anti-PD-L1 phase I clinical trials (NCT03475251, NCT03918278, NCT05200013, NCT04111445, NCT05483530) at the Macquarie University Clinical Trials Unit, Sydney, Australia. The median age of the patient cohort was 56 years (range 27–76) and 54% (7/13) were male. Five of the 13 patients (38%) had LMS, four (31%) had UPS, two (15%) had angiosarcoma, one (8%) had dedifferentiated LPS, and one (8%) had desmoplastic small round cell sarcoma (**Table 1**).

Seven of the 13 (54%) patients received PD-1 inhibitor therapy alone, and the five of the 13 (38%) patients received PD-1 inhibitor therapy in combination with: an immunoglobulin-like transcript 3 (ILT-3) inhibitor (2/5 patients, 40%), a glycoprotein-A repetition predominant (GARP) inhibitor (2/5, 40%), or a CTLA-4 inhibitor (1/5, 20%). One patient received a PD-L1 and cluster of differentiation 47 (CD47) bispecific antibody (1/13, 8%). Eleven patients (85%) received prior systemic therapy. Of these, four (36%) received one line of therapy, 4/11 (36%) received two lines, 2/11 (18%) received three lines, and 1/11 (9%) received four lines; all prior therapies were chemotherapy-based. Two of the 13 patients (15%) received anti-PD-1 inhibitor therapy as first line treatment.

For this study, clinical responders were defined as having complete response (CR), partial response (PR) or stable disease (SD) for ≥3 months as per RECIST 1.1 criteria. Seven (7/13, 54%) patients were characterised as responders. Three (3/7, 43%) of the clinical responders had prolonged stable disease for ≥ 6 months, with one patient achieving CR. Six (6/13, 46%) patients were characterised as non-responders (progressive disease (PD), as per RECIST 1.1 criteria, or with clinical progression, **Table 1**).

### Molecular features of patients with soft tissue sarcoma

**Figure 1:**
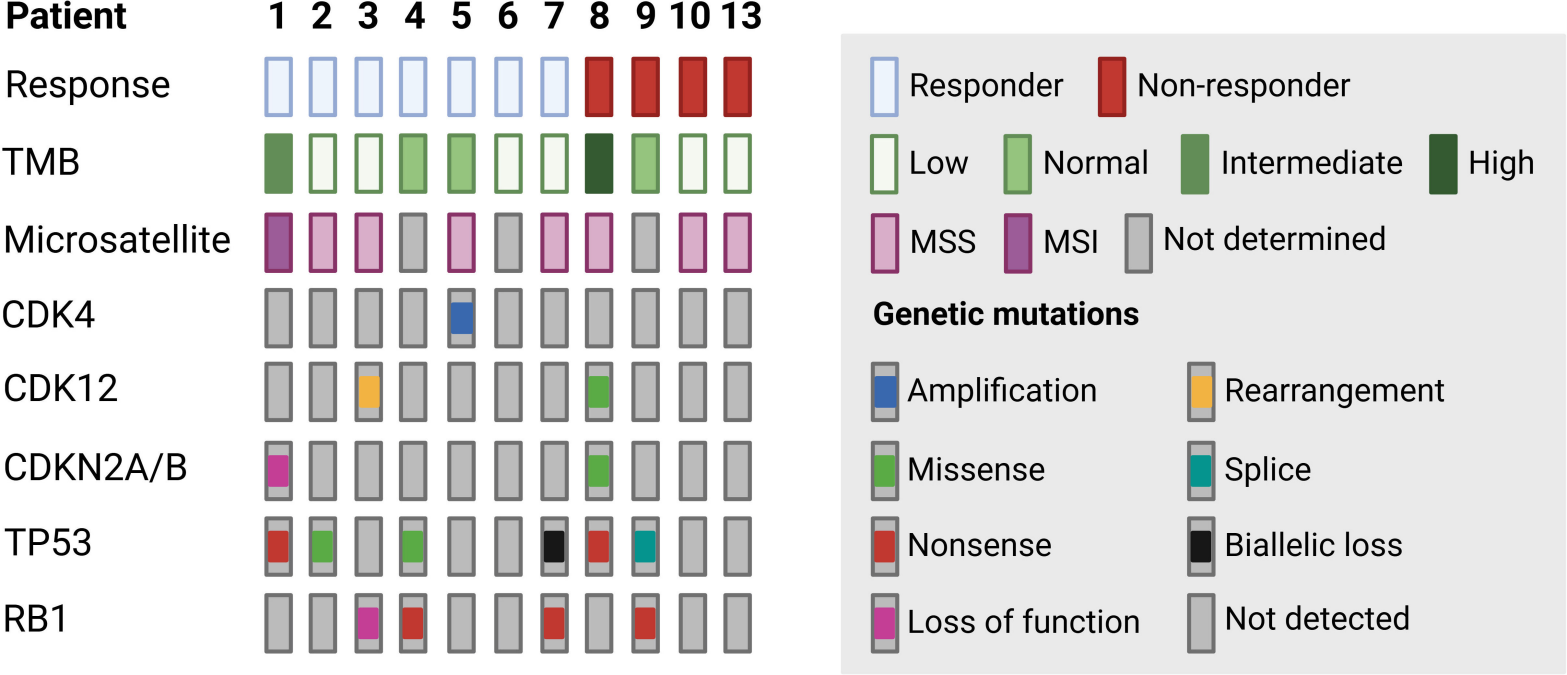
Molecular profiles of patients with soft tissue sarcoma. Mutation profiles for 11 patients (n=7 responders and n=4 non-responders) were collected as part of the Cancer Molecular Screening and Therapeutics (MoST) study. Tumour mutation burden (TMB, normal, low, intermediate or high), microsatellite status (microsatellite instability, MSI or microsatellite stability, MSS) and presence of mutations on genes (*CDK4, CDK12, CDKN2A/B, TP53* and *RB1*) implicated in ICI response and resistance are shown. Type of genetic mutations include gene amplification and rearrangement, biallelic loss, splice, missense, nonsense and loss-of-function mutations.

Mutation profiles for 11/13 patients were collected as part of the Cancer Molecular Screening and Therapeutics (MoST) study (ACTRN12616000908437). The tumour mutation burden (TMB) status was categorised for each patient as being low, normal, moderately high (i.e. intermediate), or high according to the normal range (3.8–22.8 Mut/Mb) indicated by the Molecular Tumour Board; TMB was normal for 3/11 (27%), low for 6/11 (55%), intermediate for 1/11 (9%), and high for 1/11 (9%) patients. Microsatellite status was available for eight patients and was categorised as microsatellite instability (MSI) (≥30% sites unstable) or microsatellite stability (MSS) (< 30% sites unstable); only 1/8 (12.5%) patients had MSI high while 7/8 (87.5%) patients had MSS (**Figure 1**).

Gene mutations previously implicated in ICI response including *CDK4*, *CDK12*, *CDKN2A/B*, *TP53*, and *RB1* (**Figure 1**) were also examined in the 11 patients with mutation profiles. A gene amplification leading to *CDK4* overexpression was reported in Patient 5. *CDK12* mutations were also detected, including a rearrangement in exon 12 in Patient 3 and a missense (*CDK12*^Q695*^) mutation in Patient 8. A loss-of-function mutation in *CDKN2A/B* was detected in Patient 1 while a missense mutation (*CDKN2A*^L130R^) was detected in Patient 8. *TP53* was mutated in 6/11 patients (55%), and these included nonsense mutations (in Patient 1 and 8), and missense, biallelic loss, and splice mutations in Patient 2, 4, 7 and 9, respectively. Nonsense or loss-of-function mutations in *RB1* were detected in four patients (Patient 3, 4, 7 and 9) (**Figure 1**). Overall, most patients showed variable range of TMB with no predominant driver mutations, and MSI was uncommon. Response or non-response to ICI was not associated with TMB, MSI status or genetic mutations in this cohort.

### Baseline and longitudinal analysis of complete blood counts

Complete blood counts were collected for all 13 patients at three timepoints (baseline, 3-weeks post treatment, and 6-weeks post treatment), including haemoglobin levels (g/L), white cell count (WCC, x10^9^/L), and neutrophil (x10^9^/L), lymphocyte (x10^9^/L), monocyte (x10^9^/L), eosinophil (x10^9^/L), basophil (x10^9^/L), and platelet (x10^9^/L) counts. The neutrophil-to-lymphocyte ratio (NLR), lymphocyte-to-monocyte ratio (LMR), and platelet-to-lymphocyte ratio (PLR) were also assessed for each patient at each timepoint.

**Figure 2:**
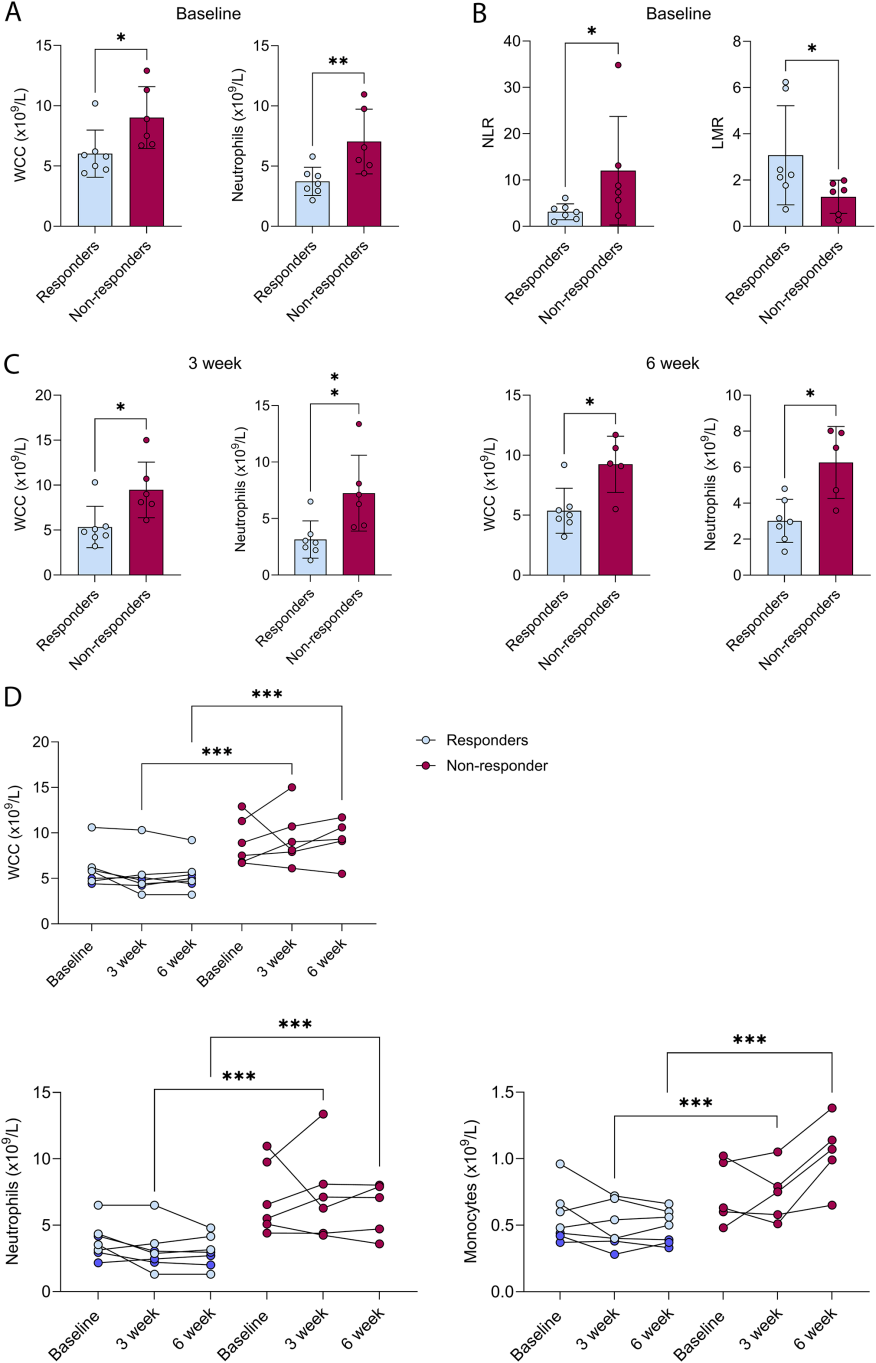
Baseline and longitudinal analysis of complete blood counts. Baseline levels of **(A)** white cell (WCC) and neutrophil counts, and **(B)** neutrophil-to-lymphocyte (NLR) and lymphocyte-to-monocyte (LMR) ratios in responders (n=7, blue) compared to non-responders (n=6, red). **(C)** Levels of WCC and neutrophil at 3- and 6-weeks post treatment initiation in responders compared to non-responders. **(D)** Levels of WCC, neutrophil and monocyte counts at baseline, 3- and 6-weeks post treatment initiation in responders (blue) compared to non-responders (red). Responders with SD ≥ 6 months are highlighted in dark blue. Values are expressed as mean ± SD of cell counts in each group, *p<0.05, **p<0.01, Mann-Whitney test; ***p<0.001, Mixed-effects analysis with Geisser-Greenhouse correction and Sidak multiple comparison.

Of all the blood count parameters assessed at baseline, only WCC, neutrophil counts, NLR and LMR were significantly different between responders and non-responders. In particular, baseline WCC was significantly lower in responders compared to non-responders (median 5.80 x10^9^/L vs 8.20 x10^9^/L, p=0.014; **Figure 2A**), and of the WCC, neutrophil counts were lower in responders compared non-responders (median 3.53 x10^9^/L vs 6.03 x10^9^/L, p=0.008, **Figure 2A**). As expected, NLR was also significantly lower in responders compared to non-responders (median 3.07 vs 8.06, p=0.035), but LMR was significantly higher (median 2.22 vs 1.53, p=0.035) (**Figure 2B**).

Complete blood counts were also analysed at 3- and 6-weeks post treatment to examine if these parameters changed over time and are indicative of treatment response. In general, treatment did not induce substantial or consistent changes in blood cell counts. Of note, both WCC and neutrophil counts remained low and were significantly lower in responders compared to non-responders at 3- and 6-weeks post treatment (**Figures 2C, D**). Interestingly, responders, particularly those with SD ≥ 6 months, showed consistently low monocyte counts at baseline, which remained low at 3- and 6-weeks post treatment, and monocyte counts were significantly lower in responders compared to non-responders at this timepoint (**Figure 2D**). To examine if these differences remained significant after adjusting for histologic subtype and treatment regimen (anti-PD-1 monotherapy vs anti-PD-1 combination), multivariable logistic regression analysis were performed. Given the small sample size and heterogeneity of STS, it was not unexpected that these associations did not remain statistically significant (**Supplementary Table 3**).

### Multiparameter immune cell profiling of baseline PBMC samples

**Figure 3:**
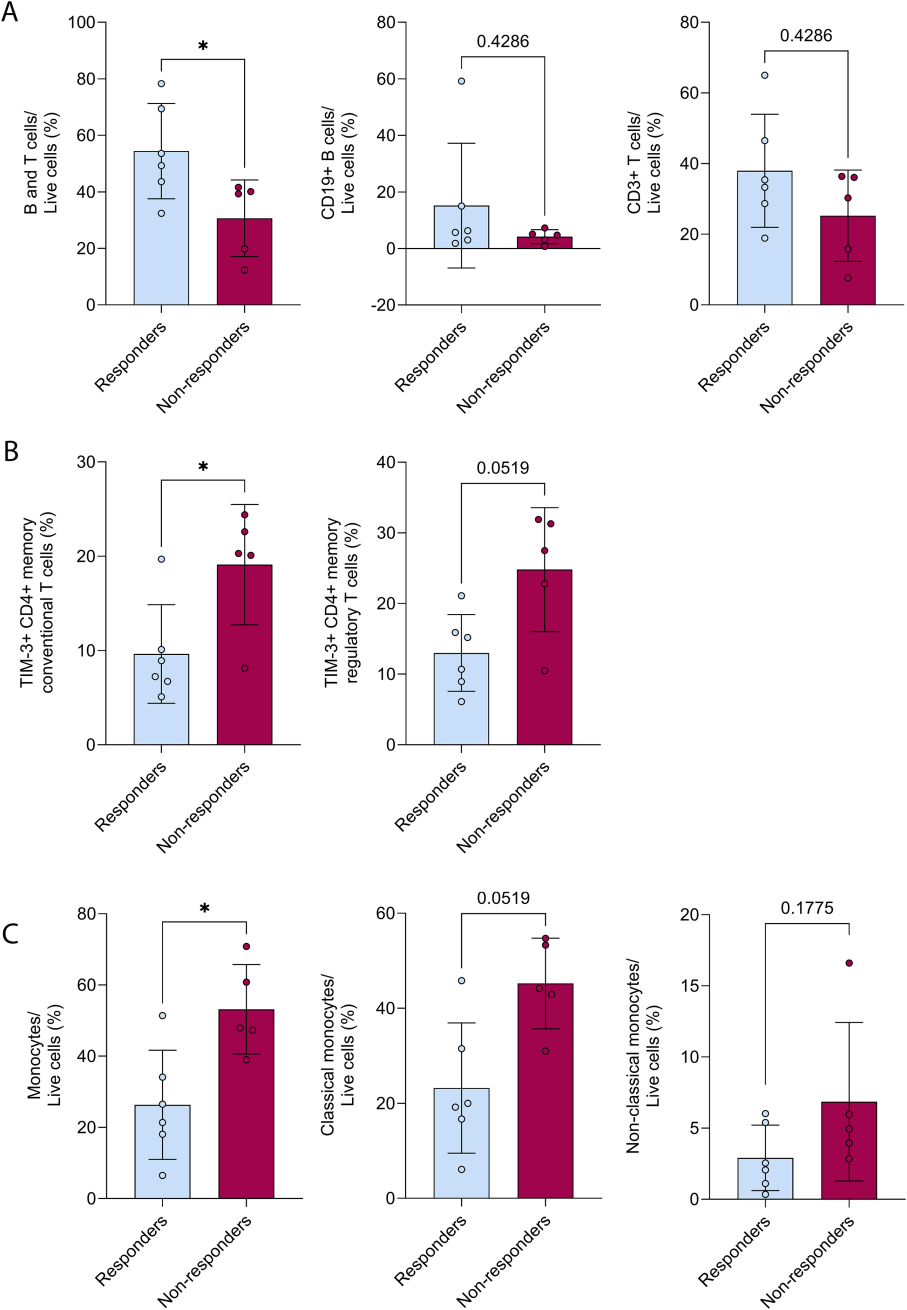
Baseline analysis of PBMCs. Baseline levels of **(A)** lymphoid immune cells; B and T cells combined or separated, as a percentage of live cells, **(B)** TIM-3+ CD4+ memory conventional and regulatory T cells frequencies, and **(C)** monocytes; classical or non-classical, as a percentage of live cells in responders (n=6, blue) compared to non-responders (n=5, red). Values are expressed as mean ± SD, *p<0.05, Mann-Whitney test.

PBMCs were available for 11 patients prior to treatment (six responders and five non-responders; PBMCs were not available for Patient 1 and 10) and these were analysed by flow cytometry to detect B and T cells, including CD8, CD4 conventional and regulatory T cells, monocytes, natural killer cells, dendritic cells and myeloid-derived suppressor cells (**Supplementary Table 2**). At baseline, the proportion of immune lymphoid cells (B and T cells combined) was significantly higher in responders compared to non-responders (median 51.45% vs 39.30%, p=0.0303; **Figure 3A**). However, closer examination of distinct lymphoid cells revealed that while B cells and T cell frequencies tended to be higher in responders, they were highly variable and not significantly different between the two groups (**Figure 3A**). Similarly, frequencies of CD8 and CD4 T cells subsets – including naïve and memory conventional and regulatory T cells – were also variable and showed no substantial differences between responders and non-responders (**Supplementary Figure 2**).

We additionally examined whether baseline expression of activating and inhibitory molecules (Ki67, LAG-3, PD-1, NKG2D, TIM-3) on distinct T cell subsets (CD8+ and CD4+ memory conventional and regulatory T cells) differed with response. Only the proportion of TIM-3+ CD4+ memory conventional T cells was significantly higher in non-responders compared to responders (median 8.08% vs 20.30%, p=0.0240); proportion of TIM-3+ CD4+ memory regulatory T cells was also higher in non-responders, but differences did not reach statistical significance (**Figure 3B**).

Of the immune myeloid cell subsets, only the proportion of monocytes was significantly lower in responders compared to non-responders (median 23.95% vs 47.90%, p=0.0122), and of these, the proportion of the classical, but not the non-classical, monocytes was numerically lower in responders compared to non-responders (median 19.60% vs 44.20%, p=0.0519; **Figure 3C**). However, multivariable logistic regression analyses accounting for histologic subtype and treatment regimen indicated that these differences did not remain statistically significant after adjustment (**Supplementary Table 3**).

### Single cell RNA sequencing of longitudinal PBMC samples from a responding patient

To uncover patterns of response to anti-PD-1, we performed flow cytometric immune profiling and single cell RNA sequencing (scRNA-seq) on longitudinal PBMC samples from a single patient (Patient 3) with matched clinical and genomic data. Patient 3 is a 55-year-old man with metastatic retroperitoneal LMS, who initially underwent laparotomy and small bowel resection, followed by pelvic exenteration. Within 6 months of surgery, a solitary liver lesion was found on routine imaging. This was resected, and the biopsy confirmed metastatic LMS while molecular testing showed *CDK12* rearrangement in exon 2, *RB1* loss-of-function mutation, low TMB and MSS. This patient was subsequently enrolled in a phase 1 clinical trial and was treated with a PD-1 inhibitor in combination with a GARP inhibitor in the first line setting.

**Figure 4:**
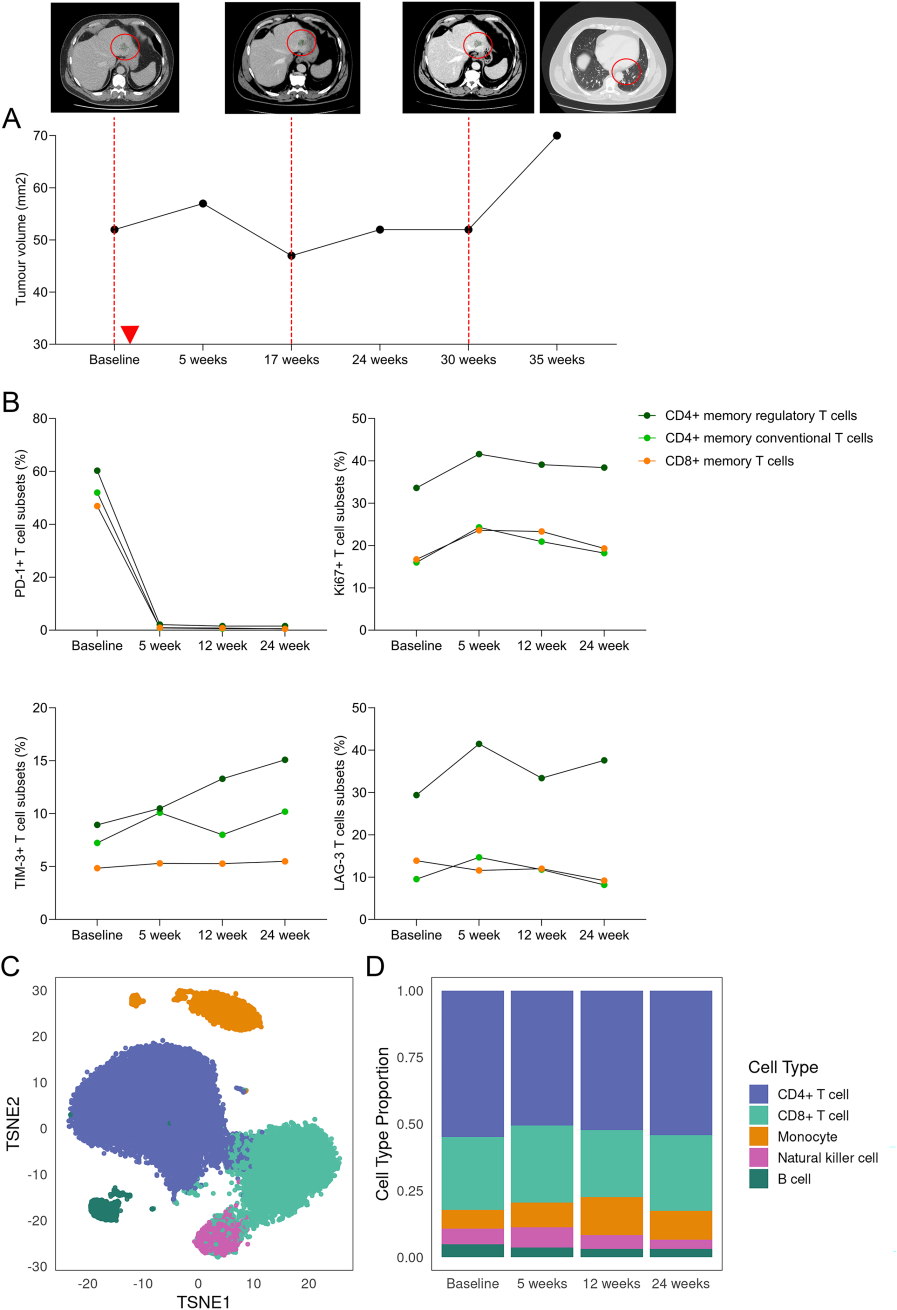
Immune cell dynamics in a patient responding to anti-PD-1 therapy. **(A)** Changes in tumour volume in a single patient (Patient 3) that exhibited stable disease on anti-PD-1 based therapy, start of therapy indicated by red arrow. Tumour volume was determined from measurement of target lesions (red circles) shown in corresponding CT scans. **(B)** Changes in expression of immune modulatory markers on CD4+ and CD8+ memory T cells at baseline and at 5-, 12- and 24-weeks post treatment. **(C)** tSNE plot showing the major cell populations identified from scRNAseq of longitudinal PBMC samples, **(D)** Frequencies of the five identified cell populations at baseline and at 5-, 12-, 24-weeks post treatment.

Prior to treatment, Patient 3 presented with metastatic lesions in the liver and renal sites, measuring 29 mm and 22 mm on CT scans, respectively. CT scan at 17-weeks post treatment showed an 18% reduction in tumour volume; this was the best response achieved by the patient and was classified as SD by RECIST1.1. Patient maintained SD for nine months on treatment. Sequential CT scans at 30-weeks post treatment initiation showed early signs of progression, with increase in target lesions and in the number and size of new lung lesions. Progressive disease was confirmed on CT scan at 35 weeks where a 37% increase in tumour volume was observed from the best response at 17 weeks (**Figure 4A**).

Flow cytometric immune profiling of PBMCs collected at baseline and at 5-, 12- and 24-weeks post treatment initiation showed considerable decrease in PD-1+ CD8+ and CD4+ memory T cells post treatment (**Figure 4B**), likely due to anti-PD-1 drug binding as it precludes binding of the anti-PD-1 antibody used for flow cytometry detection, in line with previous reports (20–22). Conversely, treatment induced transient increase in proliferation (Ki67+) of CD8+ and CD4+ memory T cells, with gradual decrease from 12- to 24-weeks post treatment. Interestingly, TIM-3+ CD4+ memory T cells, which are enriched in non-responders, increased in proportion and peaked at 24-weeks post treatment. LAG-3 expression was enriched in CD4+ memory regulatory T cells compared to CD8+ and CD4+ memory T cell subsets, and proportion of this population increased and remained elevated post treatment (**Figure 4B**).

scRNAseq was performed on the same PBMC samples (n=35,534 cells) to explore immune cell dynamics in response to treatment at single-cell resolution. Using a supervised learning methode (scClassify) (15), we identified five major cell populations: B cells, CD4+ T cells, CD8+ T cells, monocytes and natural killer cells (**Figure 4C**; **Supplementary Figure 3**). Across all four timepoints, CD4+ T cells were the most abundant (53%), followed by CD8+ T cells (median 28%) and monocytes (10%) (**Figure 4D**). Given that T cells were the predominant population, while monocytes exhibited considerable temporal variation in proportion (range 7-14%), we focused on detailed characterisation of these cell types (**Supplementary Table 4**).

**Figure 5:**
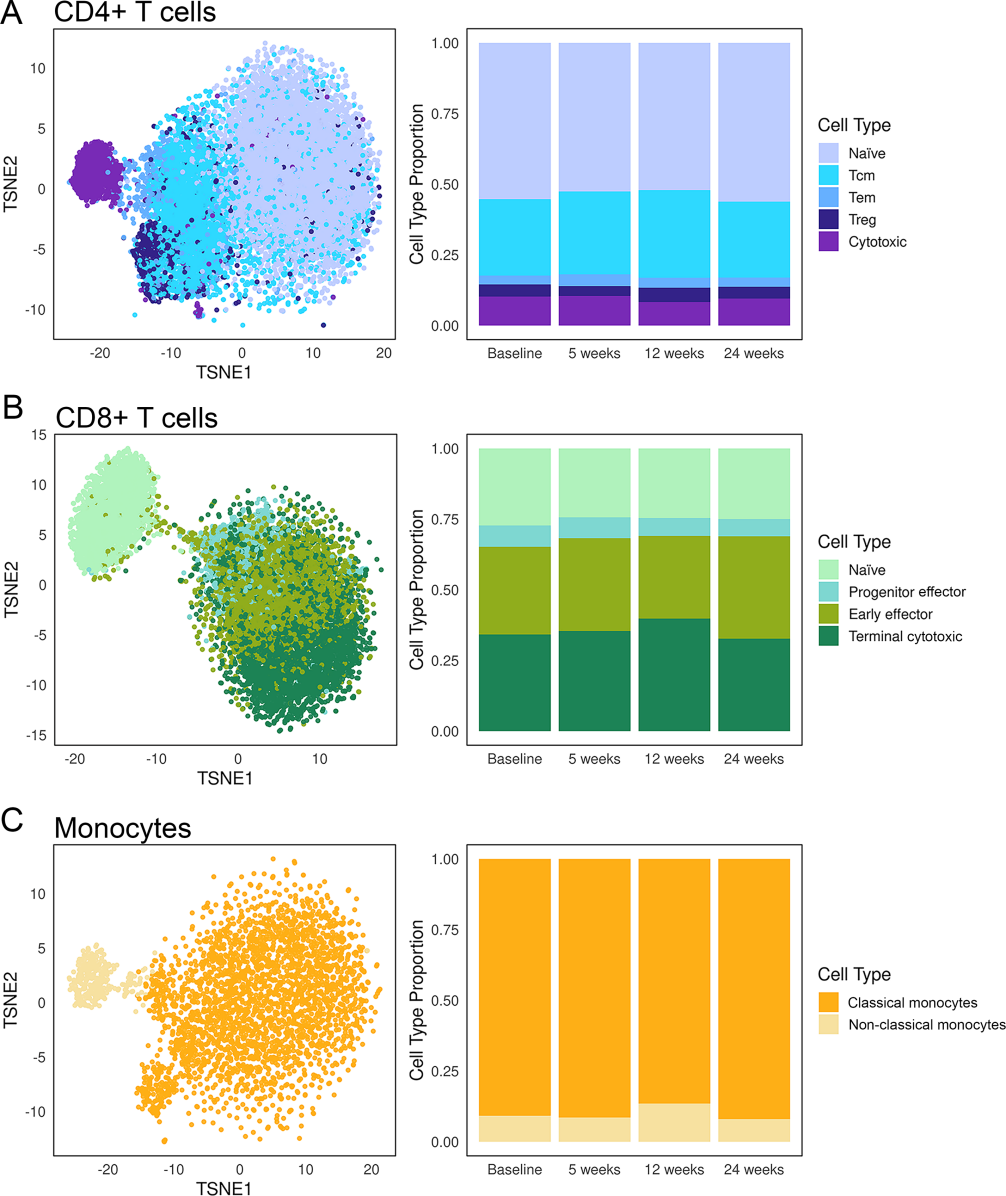
Changes in subsets of CD4+ and CD8+ T cells and monocytes post anti-PD-1 treatment in a responding patient. tSNE and barplots showing frequencies of annotated phenotypic subsets of **(A)** CD4+ T cells, **(B)** CD8+ T cells and **(C)** monocytes at baseline and at 5-, 12-, 24-weeks post treatment.

Within CD4+ T cells, five subclusters were identified and annotated as naïve, central memory, effector memory, cytotoxic and regulatory T cells. The four CD8+ T cell subclusters comprised naïve, progenitor effector, early effector and terminal cytotoxic T cells. Monocytes were further divided into classical and non-classical subsets (**Figures 5A–C**; **Supplementary Figure 4**). Among these subsets, non-classical monocytes (range 8-14%), CD4+ regulatory T cells (range 3-5%), CD4+ effector memory T cells (range 3-4%), CD4+ cytotoxic T cells (range 8-10%) and CD8+ progenitor effector T cells (range 6-8%), showed the most pronounced shifts in proportion over the course of treatment (**Supplementary Table 4**). Notably, changes in proportion of non-classical monocytes and CD8+ terminal cytotoxic T cells parallel changes in tumour burden on CT scan of the patient. For instance, proportion of these cells increased 12 weeks post-treatment (from 9% to 14% for non-classical monocytes and from 34% to 40% for CD8+ terminal cytotoxic T cells compared to baseline), which preceded the best response on CT scan at 17 weeks. Additionally, frequencies of these cell subsets gradually decreased 24 weeks post-treatment, and this preceded signs of early progression at 30 weeks (**Figure 5**).

**Figure 6:**
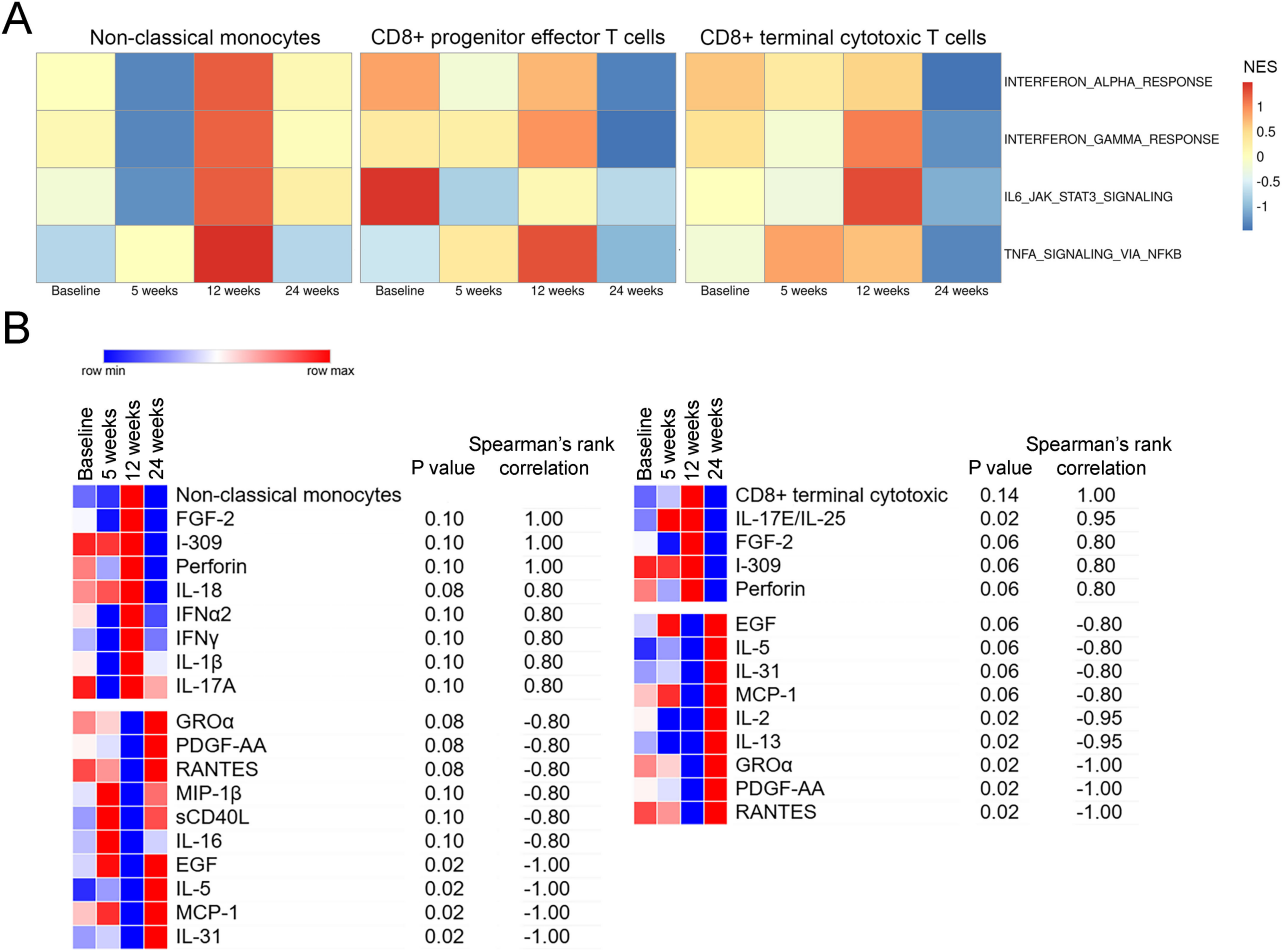
Single sample Gene Set Enrichment Analysis (ssGSEA) in distinct immune cell phenotypes and plasma protein profiling following anti-PD-1 treatment in a responding patient. **(A)** ssGSEA of the Hallmark inflammatory gene signatures (Interferon Alpha and Interferon Gamma Response, IL6 JAK STAT3 and TNFA Signalling) in non-classical monocytes, CD8+ progenitor effector and terminal cytotoxic T cells. Comparison separated by timepoints and normalised enrichment scores (NES) shown. **(B)** Plasma protein correlation with non-classical monocytes and CD8+ terminal cytotoxic T cells. Spearman’s correlation analyses were performed in Morpheus and p values and Spearman’s correlation coefficient shown.

We additionally performed single sample Gene Set Enrichment Analysis (ssGSEA) using Hallmark signatures and cytokine profiling of matched longitudinal plasma samples from Patient 3 to infer functional activity of these immune cell subsets over time (**Figure 6**; **Supplementary Figure 5**). Non-classical monocytes showed enrichment of Hallmark inflammatory gene sets (e.g., Hallmark interferon alpha and interferon gamma response, IL6 JAK STAT3 and TNFA signalling) at 12 weeks post treatment compared to baseline, coinciding with their increased proportion; however, these signatures were no longer enriched at 24 weeks. Similarly, CD8+ terminal cytotoxic and progenitor effector T cells also showed enrichment of inflammatory gene sets at 12 weeks, which diminished by 24 weeks post treatment (**Figure 6A**). Frequency of non-classical monocytes strongly correlated with circulating FGF-2, I-309, perforin, IL-18, IFNα2, IFNγ, IL-1β and IL-17A levels (Spearman’s correlation coefficient r = 0.8-1), suggesting association with a functionally activated immune milieu. Moreover, proportion of CD8+ terminal cytotoxic T cells showed similar positive correlations with FGF-2, I-309, perforin and IL-17E (Spearman’s correlation coefficient r = 0.8-1, **Figure 6B**), supporting their cytotoxic effector functions. Altogether, these findings highlight a pattern in which these immune cell subsets show heightened and coordinated inflammatory and cytotoxic activity at 12 weeks, prior to best response on CT scan, followed by a decline at 24 weeks, preceding early signs of disease progression.

## Discussion

In this study, we provide evidence that peripheral immune cell profiles are associated with response to anti-PD-1–based therapy in STS. Specifically, lower circulating neutrophil and monocyte counts, higher lymphoid cell proportions, and reduced proportion of TIM-3-expressing CD4+ memory T cells at baseline distinguished responders from non-responders. Additionally, longitudinal single-cell RNA sequencing of a patient with prolonged stable disease revealed dynamic shifts in proportion and activity of non-classical monocytes and CD8+ T cell subsets that paralleled radiological response and disease progression. Collectively, these findings highlight the potential of blood-based immune biomarkers to inform therapeutic stratification and real-time monitoring in STS.

Our findings are consistent with prior studies demonstrating the relevance of systemic inflammation markers, such as NLR and LMR, in predicting ICI response across multiple tumour types. For instance, in a retrospective cohort of 1714 patients with 16 different cancer types including melanoma, non-small cell lung cancer and renal cancer, higher baseline NLR is significantly associated with poorer PFS and OS, and lower rates of response to ICI therapy (23). In contrast, higher baseline LMR is associated with better PFS, OS and disease control rate in 4322 patients with different cancer types treated with ICI (24). Nevertheless, predictive ICI biomarkers in STS remain poorly defined. Although several haematological indices have been shown to prognosticate STS patients (reviewed in (12)), few studies have demonstrated their association with ICI response. Additionally, tumour-based biomarkers such as TMB and microsatellite status have shown limited clinical utility due to their low prevalence in STS (25), and this is similarly reflected in our cohort. Recently, correlative analysis from the SARC028 study has shown that certain immune cell types prior to treatment are associated with response to anti-PD-1 (26). Taken together, these findings highlight the need to identify reliable biomarkers of ICI response in STS, and support blood-based immune profiling as a more practical, valuable and informative approach to guide patient selection and treatment monitoring.

The enrichment of neutrophils and monocytes in non-responders at baseline and throughout treatment supports the concept that myeloid-driven immunosuppression diminishes ICI response in STS (27, 28). Conversely, higher circulating lymphoid frequencies may reflect a more immune-permissive environment (29), favouring sustained effector T cell responses and tumour control under PD-1 blockade. Indeed, activated T cells were enriched in tumour biopsies of STS patients who responded to anti-PD-1 therapy (26). Comprehensive profiling of T cell subsets further revealed augmented TIM-3 expression on lymphocytes, particularly CD4+ memory T cells, in non-responders, suggesting that more granular sub-phenotyping can further distinguish ICI outcomes. The increased proportion of TIM-3-expressing CD4+ memory T cells in non-responders aligns with prior studies linking TIM-3 expression to T cell exhaustion and resistance to PD-1 blockade (30), and higher TIM-3 expression on CD8+ T cells correlating with poorer disease-free survival in STS (31).

TIM-3 is recognised as a marker of late-stage or terminal T-cell exhaustion (32) and is frequently co-expressed with PD-1 in dysfunctional or exhausted T cells (33, 34). Upregulation of TIM-3 has been proposed as a compensatory inhibitory pathway following PD-1 blockade in several cancer types (35–37), contributing to adaptive resistance. The enrichment of TIM-3+ CD4+ memory T cells in non-responders may therefore reflect an exhausted or dysfunctional immune state that constraints effective anti-tumour activity despite PD-1 blockade. Although we did not directly assess the mechanisms governing TIM-3 expression, these studies highlight TIM-3 as a potential mediator of resistance to PD-1 blockade in STS. Moreover, given that TIM-3+ T cells comprise approximately 20% of circulating T cells in this cohort, it is plausible that combination anti-PD-1 and anti-TIM-3 therapy could provide clinical benefit in STS by targeting non-redundant inhibitory pathways. Supporting this rationale, early phase trials of this combination have shown preliminary anti-tumour activity, with partial response observed in 6% of patients with solid tumours (38) and overall response rates of 42.9% in immunotherapy-naïve melanoma patients (39).

Our case study and single-cell analyses provide additional insights into the temporal dynamics of the peripheral immune response to anti-PD-1 therapy. In particular, expansion of non-classical monocytes and terminal cytotoxic CD8+ T cells coincided with best radiological response, while their subsequent decline preceded disease progression. Enrichment of oxidative phosphorylation and inflammatory pathways in the non-classical monocytes suggests a transiently activated phenotype that may support T cell priming and effector function. Indeed, cytokine-producing non-classical monocytes have been shown to enhance CD8+ T effector cell functions (40), and to improve efficacy of ICIs in a mouse model of colon cancer (41). Although overall monocyte abundance was greater in ICI non-responders, single-cell transcriptome analysis enabled granular distinction between the monocytes subsets and revealed that non-classical monocytes were associated with ICI response. This suggests that the balance between monocyte subsets may be critical, with a higher ratio of non-classical to classical monocytes favouring response while predominance of classical monocytes may be associated with progression. Additionally, terminally-differentiated CD8+ T cells, recognised as highly potent effector cells with elevated levels of cytotoxic molecules such as granzyme B and perforin (42), displayed fluctuations that mirrored treatment response. The dynamic shifts of this cytotoxic subset in circulation, together with enrichment of inflammatory activity, suggest they are crucial for tumour control during PD-1 blockade. While direct functional assays were not performed, plasma cytokine profiling provided orthogonal evidence of immune activity, with abundance of non-classical monocytes and terminal cytotoxic CD8+ T cells correlating with a pro-inflammatory and cytotoxic cytokine milieu, including interferons, inflammasome-associated cytokines and perforin. These associations support a functional link between these circulating immune cells and systemic immune activation, although causality cannot be inferred. Taken together, these findings suggest that dynamic changes in specific circulating immune subsets may serve as early indicators of treatment efficacy or disease progression, while revealing cell types potentially critical for mediating ICI response. Nonetheless, direct functional interrogation of these subsets will be required to define their mechanistic roles, and validation in larger, multicentre cohorts is needed to establish their utility as circulating biomarkers.

Our exploratory study is also limited by the small cohort size, reflecting the rarity of STS and limited access to patients treated with ICIs, as well as by histological heterogeneity across included subtypes, which constrains statistical power and generalisability. Although responders were confined to STS subtypes previously reported to benefit from ICI, namely undifferentiated pleomorphic sarcoma and dedifferentiated liposarcoma (8), these findings should be interpreted cautiously. Additionally, molecular profiling was incomplete for some patients, further limiting integrated analyses. Despite these limitations, this study provides proof-of-concept that peripheral immune correlates can be detected in STS and may have predictive value. Peripheral immune monitoring is minimally invasive and could complement tissue-based biomarkers to enable patient stratification and early identification of non-responders. Importantly, validation in larger, ideally multicentre, cohorts will be required to enable adequately powered multivariable analyses and to definitively establish the independent prognostic and predictive value of these biomarkers.

In conclusion, this work identifies baseline and dynamic features of circulating immune cells associated with ICI efficacy in STS. Although these findings warrant validation in larger, multi-centre cohorts, they provide strong rationale for incorporating peripheral immune monitoring into future clinical trials to improve patient stratification and accelerate biomarker development in this rare and challenging disease.

## Data Availability

Single-cell RNA sequencing data is deposited to the Sequence Read Archive under the submission code PRJNA1421050. The code used in the single-cell RNA sequencing data analysis is provided as part of a git repository and can be accessed from https://github.com/SydneyBioX/soft-tissue-sarcoma-project-pbmc-scRNAseq. Further inquiries can be directed to the corresponding author.
