# Supplementary material for: Distinct immunologic patterns of response and resistance to anti-PD-1/PD-L1-based immunotherapy in patients with soft tissue sarcoma
Source: Front Immunol. 2026 Mar 5;17:1783216. doi: 10.3389/fimmu.2026.1783216 (PMC12999908; doi:10.3389/fimmu.2026.1783216)
Supplement: Supplementary file 1 [file Presentation1.pdf]

Supplementary Table 1: Antibodies used in immune cell profiling of PBMCs by flow cytometry

Antibody	Clone	Cat No	Company	Dilution used
CD1c PE-Cy7	L161	331516	BioLegend	1/40
CD3 BUV395	UCHT-1	563646	BD Horizon	1/20
CD3 BUV737	UCHT-1	612750	BD Horizon	1/100
CD4 FITC	SK3	344604	BioLegend	1/200
CD8 V500	SK1	561617	BD Horizon	1/100
CD11b BUV496	ICRF44	741138	BD OptiBuild	1/50
CD14 V500	M5E2	561391	BD Horizon	1/80
CD15 BV786	W6D3	741013	BD OptiBuild	1/200
CD16 AF700	3G8	557920	BD Pharmingen	1/100
CD19 BUV496	SJ25C1	612938	BD Horizon	1/20
CD19 BUV737	SJ25C1	564303	BD Horizon	1/100
CD33 BV421	WM-53	562854	BD Horizon	1/50
CD45RA BUV737	HI100	564442	BD Horizon	1/100
CD56 PE	REA196	130-113-312	Miltenyi	1/50
CD66b FITC	REA306	130-104-413	Miltenyi	1/40
CD141 PE Dazzle 594	M80	344120	BioLegend	1/40
CD223 (LAG-3) PE	REA351	130-105-452	Miltenyi	1/10
CD279 (PD-1) BV421	EH12.1	562516	BD Horizon	1/40
CD314 (NKG2D) PE-Cy7	1D11	562365	BioLegend	1/50
CD366 (TIM-3) BV786	7D3	742857	BD OptiBuild	1/20
Fc block	Fc1	564220	BD Pharmingen	1/200
Fixable Near-IR Dead Cell Stain		L34976	Thermo Fisher Scientific	1/100
FOXP3 PECF594	236A/E7	563955	BD Horizon	1/20
HLA-DR, DP, DQ BUV395	Tu39	740302	BD OptiBuild	1/200
Ki67 APC	20Raj1	17-5699-42	Thermo Fisher Scientific	1/200

Supplementary Table 2: Flow cytometry gating

Cell subset	Phenotype
General gating	Non-debris; Single cells; Time gate; LiveDead (neg)
B cells	CD3 (neg); SCC-A (low); CD19 (pos)
CD8 T cells	CD3 (pos); SCC-A (low); CD4 (neg); CD8 (pos)<br>Naïve: CD45RA (pos)<br>Memory: CD45RA (neg)
CD4 T conventional cells (Tconv)	CD3 (pos); SCC-A (low); CD8 (neg); CD4 (pos); FOXP3 (neg)<br>Naïve: CD45RA (pos)<br>Memory: CD45RA (neg)
CD4 T regulatory (Treg)	CD3 (pos); SCC-A (low); CD8 (neg); CD4 (pos); FOXP3 (pos)<br>Naïve: CD45RA (pos)<br>Memory: CD45RA (neg)
Monocytes	CD3 (neg); CD19 (neg); CD11b (pos); CD14 (pos); CD66b (low); CD15 (low)<br>Classical: CD16 (neg)<br>Non-classical: CD16 (pos)
Natural killer cells	CD3 (neg); CD19 (neg); CD11b (pos); CD14 (neg)<br>Subsets:<br>CD56 (pos); CD16 (pos)<br>CD56 (pos); CD16 (neg)<br>CD56 (neg); CD16 (pos)
Classical dendritic cells 1 (cDC1)	CD3 (neg); CD19 (neg); CD11b (pos); CD14 (neg); CD56 (neg); CD16 (neg); CD141 (pos); HLA-DR (pos)
Classical dendritic cells 2 (cDC2)	CD3 (neg); CD19 (neg); CD11b (pos); CD14 (neg); CD56 (neg); CD16 (neg); CD1c (pos); HLA-DR (pos)
Myeloid-derived suppressor cells	CD3 (neg); CD19 (neg); CD11b (pos); CD14 (neg); CD56 (neg); CD16 (neg); HLA-DR (neg); CD33 (pos)

Supplementary Table 3: Multivariate analysis of ICI response adjusting for STS subtypes and treatment regimen

Variable	Odds ratio	95% confidence interval	P value
**Baseline complete blood counts**			
**WCC**	0.673	0.309-1.465	0.318
**Neutrophils**	0.528	0.17-1.643	0.27
**NLR**	0.921	0.763-1.112	0.393
**LMR**	1.859	0.716-4.828	0.203
**Baseline peripheral blood mononuclear cells**			
**B and T cells**	1.066	0.972-1.169	0.178
**TIM3+ CD4 memory conventional T cells**	0.718	0.474-1.087	0.117
**Monocytes**	0.927	0.837-1.026	0.145

Abbreviations: WCC, white cell count; NLR, neutrophil to lymphocyte ratio; LMR, lymphocyte to monocyte ratio

All variables with $p<0.05$ in univariate analyses were included in the multivariate model. Multivariable logistic regression was performed, adjusting for covariates (STS subtype and treatment regimen).

Supplementary Table 4: Frequencies of immune cell subsets identified from scRNAseq.

Immune cell	Baseline	5 weeks	12 weeks	24 weeks
**A. Major immune cell subsets as a proportion of total PBMCs**				
**B cell**	4.83	3.71	3.10	3.09
**CD4+ T cell**	54.98	50.60	52.33	54.17
**CD8+ T cell**	27.29	28.90	28.42	1.13
**Monocyte**	6.99	9.31	14.22	10.91
**Natural killer cell**	5.92	7.49	5.25	3.41
**B. Distinct immune phenotypes as a proportion of immune cell subsets**				
**CD4+ T cells**				
**Naive**	55.28	52.58	52.09	56.19
**Central memory**	27.03	29.33	30.97	26.86
**Effector memory**	3.18	4.18	3.55	3.25
**Cytotoxic**	10.26	10.46	8.31	9.54
**Regulatory**	4.24	3.46	5.08	4.17
**CD8+ T cells**				
**Naive**	27.25	24.36	24.61	24.96
**Early effector**	31.04	32.81	29.22	36.18
**Progenitor effector**	7.56	7.4	6.34	6.13
**Terminal cytotoxic**	34.15	35.43	39.83	32.72
**Monocytes**				
**Classical**	90.81	91.43	86.49	91.97
**Non-classical**	9.19	8.57	13.51	8.03

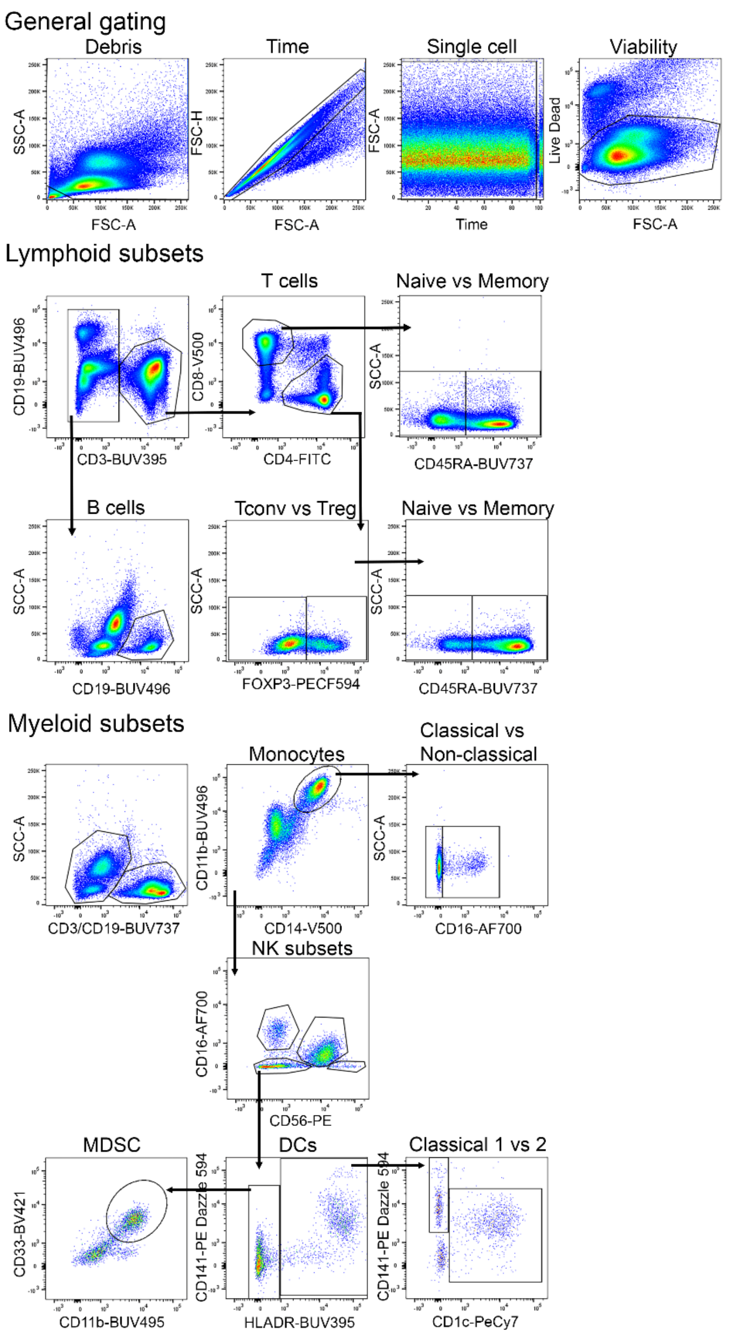

Supplementary Figure 1: Flow cytometry gating strategy to identify lymphoid and myeloid immune cell subsets.
General gating: left to right; debris exclusion gate, time gate to exclude electronic noise, single cell gate to exclude doublets, viability gate to exclude dead cells.
Lymphoid subsets: B cells (CD3-, SSC-A low, CD19+), T cell subsets (CD3+, CD8+ or CD4+), CD4+ T conventional (Tconv; FOXP3-) vs regulatory (Treg; FOXP3+), naïve (CD45RA+) vs memory (CD45RA-). CD8 and CD4 T cells subsets were further analysed for expression of inhibitory (PD-1, LAG-3, and TIM-3) and activation (Ki67 and NKG2D) markers.
Myeloid subsets: Myeloid cells (CD3-, CD19-), monocytes (CD11b+, CD14+): classical (CD16-) vs non-classical (CD16+). Natural killer cell subsets (CD56+, CD16+; CD56+, CD16-; CD56-, CD16+), dendritic cells (DCs; CD56-, CD16-, HLA-DR+): classical 1 (CD141+) vs classical 2 (CD1c+), myeloid-derived suppressor cells (CD56-, CD16-, HLA-DR-, CD33+).

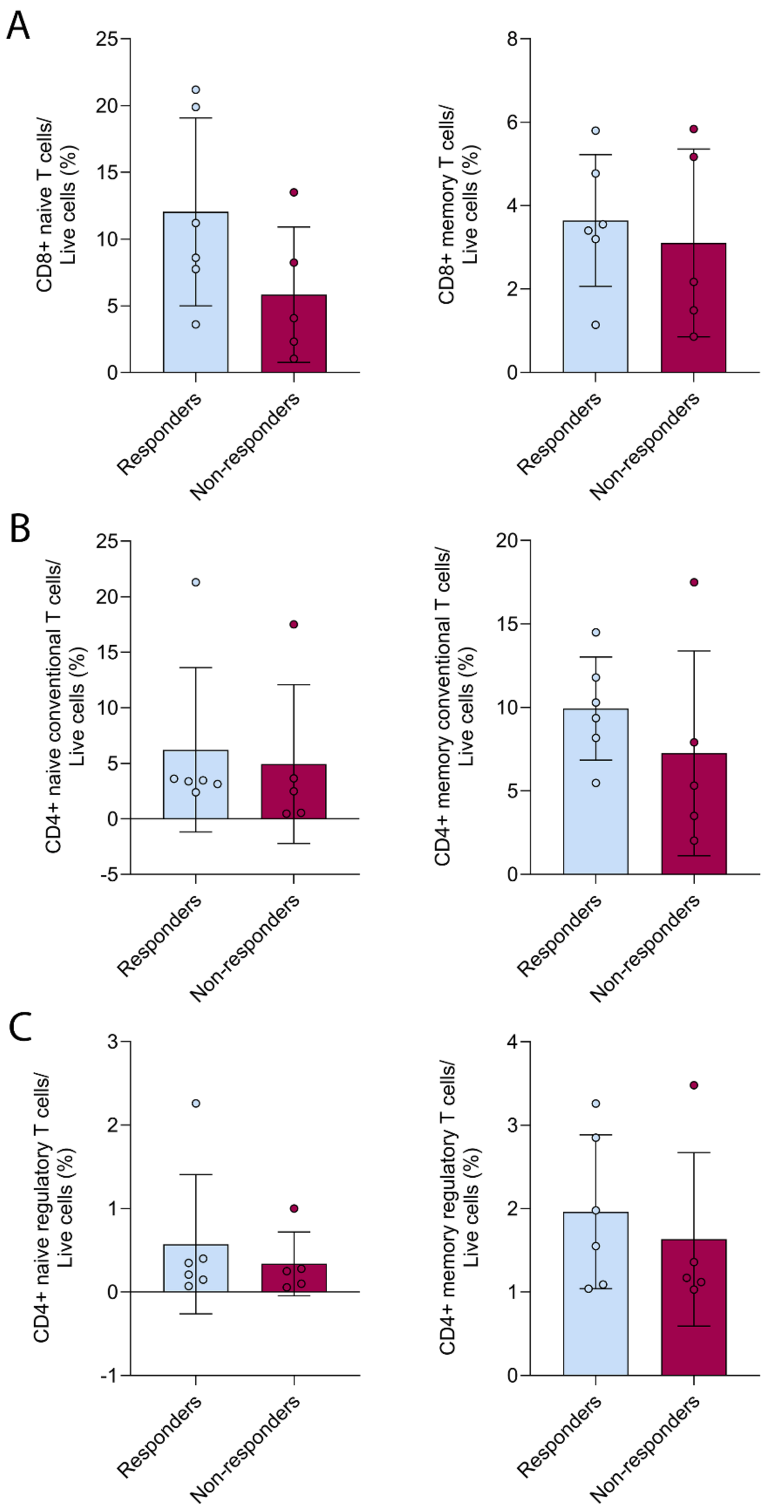

Supplementary Figure 2: Baseline analysis of T cell subsets.
Baseline levels of (A) CD8+ naive and memory T cells, (B) CD4+ naive and memory conventional T cells, and (C) CD4+ naive and memory regulatory T cells. Cell frequencies expressed as a percentage of live cells in responders (n=6, blue) compared to non-responders (n=5, red). Values are expressed as mean ± SD.

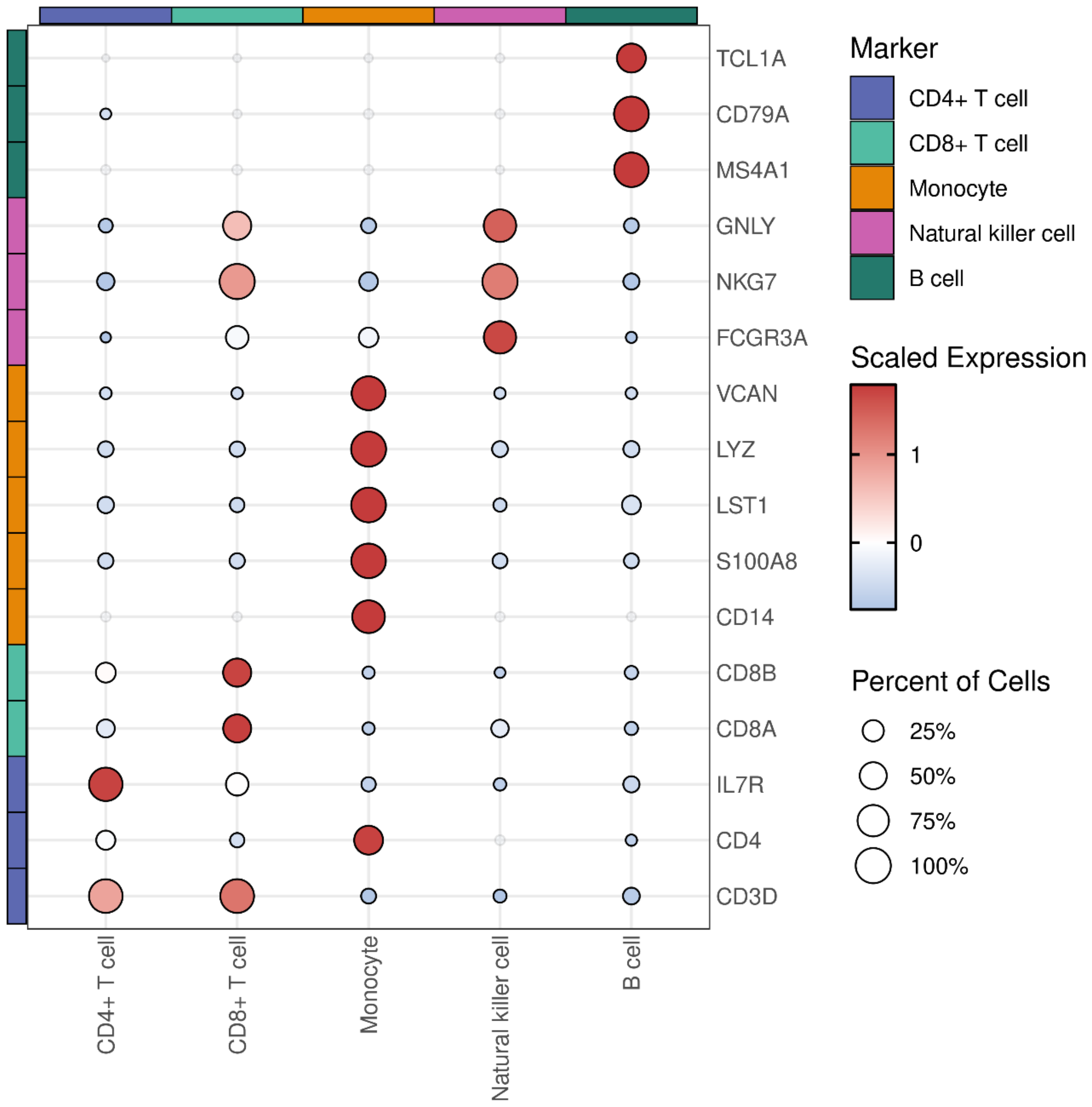

Supplementary Figure 3: Annotation of major immune cell subsets
Heatmap showing marker genes used to identify major immune cell subsets (B cells, CD4+ T cells, CD8+ T cells, monocytes and natural killer cells) in PBMC samples.

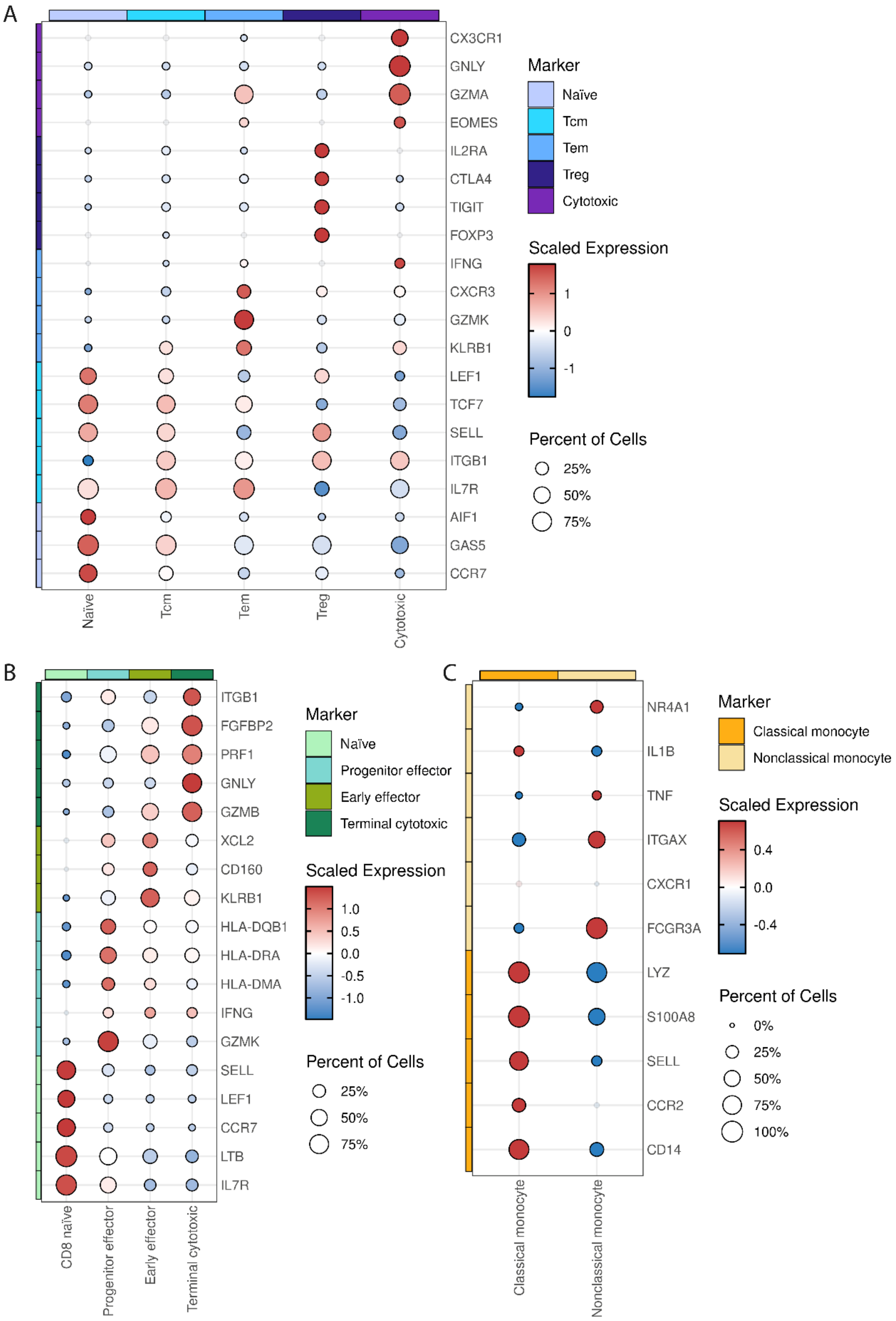

Supplementary Figure 4: Annotation of distinct immune CD4+ T cell, CD8+ T cells and monocyte phenotypes

Heatmap showing marker genes used to identify (A) CD4+ T cell (naïve, central memory, effector memory, cytotoxic and regulatory), (B) CD8+ T cell (naïve, progenitor effector, early effector and terminal cytotoxic) and (C) monocyte (classical, non-classical) subsets in PBMC samples.

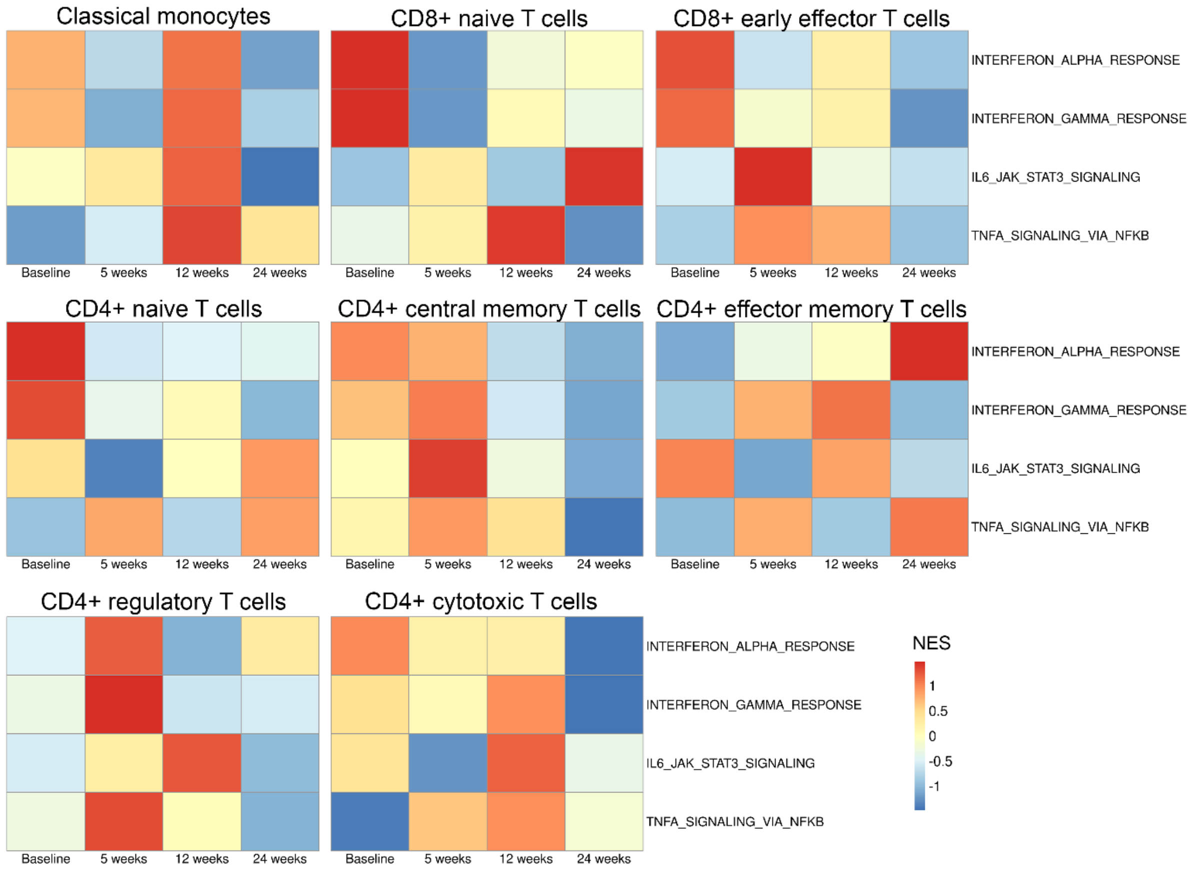

Supplementary Figure 5: Single sample Gene Set Enrichment Analysis (ssGSEA) in distinct immune cell phenotypes following anti-PD-1 treatment

ssGSEA of the Hallmark inflammatory gene signatures (Interferon Alpha and Interferon Gamma Response, IL6 JAK STAT3 and TNFA Signalling) in non-classical monocytes, CD8+ progenitor effector and terminal cytotoxic T cells. Comparison separated by timepoints and normalised enrichment scores (NES) shown.
